# Driving pressure is not associated with mortality in mechanically ventilated patients without ARDS

**DOI:** 10.1186/s13054-019-2698-9

**Published:** 2019-12-27

**Authors:** Michael J. Lanspa, Ithan D. Peltan, Jason R. Jacobs, Jeffrey S. Sorensen, Lori Carpenter, Jeffrey P. Ferraro, Samuel M. Brown, Jay G. Berry, Raj Srivastava, Colin K. Grissom

**Affiliations:** 1 Division of Pulmonary and Critical Care, Intermountain Medical Center, Shock Trauma ICU, 5121 S. Cottonwood Street, Murray, UT 84107 USA; 2 Division of Pulmonary and Critical Care, University of Utah, Salt Lake City, UT USA; 3 Intermountain Healthcare, Salt Lake City, UT USA; 4 Department of Biomedical Informatics, University of Utah, Salt Lake City, UT USA; 5 Division of General Pediatrics, Harvard Medical School, Boston, MA USA; 6 Healthcare Delivery Institute, Intermountain Healthcare, Salt Lake City, UT USA; 7 Division of Inpatient Medicine, Department of Pediatrics, University of Utah and Primary Children’s Hospital, Salt Lake City, UT USA

**Keywords:** Driving pressure, Lung protective ventilation, Low tidal volume ventilation, ARDS, Respiratory compliance

## Abstract

**Background:**

In patients with acute respiratory distress syndrome (ARDS), low tidal volume ventilation has been associated with reduced mortality. Driving pressure (tidal volume normalized to respiratory system compliance) may be an even stronger predictor of ARDS survival than tidal volume. We sought to study whether these associations hold true in acute respiratory failure patients without ARDS.

**Methods:**

This is a retrospectively cohort analysis of mechanically ventilated adult patients admitted to ICUs from 12 hospitals over 2 years. We used natural language processing of chest radiograph reports and data from the electronic medical record to identify patients who had ARDS. We used multivariable logistic regression and generalized linear models to estimate associations between tidal volume, driving pressure, and respiratory system compliance with adjusted 30-day mortality using covariates of Acute Physiology Score (APS), Charlson Comorbidity Index (CCI), age, and PaO_2_/FiO_2_ ratio.

**Results:**

We studied 2641 patients; 48% had ARDS (*n* = 1273). Patients with ARDS had higher mean APS (25 vs. 23, *p* < .001) but similar CCI (4 vs. 3, *p* = 0.6) scores. For non-ARDS patients, tidal volume was associated with increased adjusted mortality (OR 1.18 per 1 mL/kg PBW increase in tidal volume, CI 1.04 to 1.35, *p* = 0.010). We observed no association between driving pressure or respiratory compliance and mortality in patients without ARDS. In ARDS patients, both ΔP (OR1.1, CI 1.06–1.14, *p* < 0.001) and tidal volume (OR 1.17, CI 1.04–1.31, *p* = 0.007) were associated with mortality.

**Conclusions:**

In a large retrospective analysis of critically ill non-ARDS patients receiving mechanical ventilation, we found that tidal volume was associated with 30-day mortality, while driving pressure was not.

## Introduction

Mechanical ventilation with high tidal volumes may damage the lung through alveolar overdistension (volutrauma and barotrauma) and by releasing inflammatory cytokines (biotrauma) into the systemic circulation [1–3]. Lung-protective ventilation limits tidal volume and distending pressure on the alveolus in order to prevent mechanical ventilation-induced lung injury and improves survival in patients with acute respiratory distress syndrome (ARDS). In a randomized clinical trial performed by the National Institutes of Health, National Heart Lung and Blood Institute (NIH/NHLBI) ARDS Network, mortality in patients with ARDS was decreased with volume control ventilation using tidal volume of 6 mL/kg versus 12 mL/kg predicted body weight (PBW) and targeting a plateau pressure (P_PL_) of ≤ 30 cm H_2_O versus ≤ 50 cm H_2_O [2]. Consequently, professional societies have recommended lung-protective ventilation strategies for patients with ARDS [4].

Lung-protective ventilation in patients without ARDS may decrease the development of ARDS, pulmonary complications, and mortality [3, 5–8]. A meta-analysis of mechanically ventilated non-ARDS patients demonstrated that a mean tidal volume of 6.5 mL/kg versus 10.6 mL/kg PBW resulted in less development of acute lung injury or ARDS, fewer pulmonary infections, and lower mortality [6]. Further evidence of benefit from lung-protective ventilation is supported by a systematic review and patient-level analysis that demonstrated a lower incidence of ARDS and fewer pulmonary complications in non-ARDS patients treated with a tidal volume of < 7 mL/kg PBW [7]. However, lung-protective ventilation may not be optimal for all non-ARDS patients. In non-ARDS patients, the functional lung volume is greater, and lung-protective ventilation may cause ventilation-perfusion mismatch, alveolar hypoventilation, and patient-ventilator dyssynchrony [5]. In contrast to the aforementioned studies, a recent prospective randomized clinical trial found no benefit to targeting a tidal volume of 6 mL/kg PBW versus 10 mL/kg PBW while keeping P_PL_ below 25 cm H_2_O in non-ARDS patients [9].

In patients with ARDS, tidal volume normalized to respiratory system compliance (driving pressure) may be a better predictor of survival than tidal volume scaled to normal lung volume using PBW determined by height and sex [10, 11]. Driving pressure (∆P) is the difference between P_PL_ and positive end expiratory pressure (PEEP) and can be influenced by changes in tidal volume or PEEP, or respiratory system compliance. Lowering tidal volume decreases ∆P. Raising PEEP can also decrease ∆P if significant lung recruitment occurs. Despite the association of ∆P and mortality in patients with ARDS, this association is less clear in non-ARDS patients. One prior single-center study of non-ARDS patients suggested lack of an association between ∆P and mortality, but this finding has not been externally validated in a larger population [12]. Some authors suggest that ∆P may be a goal in itself for ARDS management, using ∆P as a threshold for safety to decrease ventilator-induced lung injury [13].

To assess the association between tidal volume and ∆P on mortality in non-ARDS patients, we analyzed mechanically ventilated patients in intensive care units (ICUs) at Intermountain Healthcare, the largest healthcare system in the Intermountain West, comprising 23 hospitals in Utah and Idaho. Our objective was to determine whether tidal volume and ∆P are associated with mortality in mechanically ventilated patients without ARDS. We hypothesized that increased tidal volume and increased ∆P are both associated with increased mortality in non-ARDS patients.

## Methods

### Design

We conducted a retrospective cohort study of mechanically ventilated patients in adult medical, surgical, trauma, and cardiac ICUs at 12 hospitals in Utah and Idaho over a 2-year period from January 1, 2014, to December 31, 2015. This study was approved with waiver of informed consent by the Intermountain Healthcare Institutional Review Board.

### Population

We included patients at least 18 years old who had initiation of volume-control or adaptive pressure control (APC) using pressure-regulated volume control (PRVC) mechanical ventilation in the emergency department or ICU and who were mechanically ventilated for at least 24 h. We excluded patients whose initial mode of mechanical ventilation was pressure control, airway pressure release ventilation, pressure support (PS), or continuous positive airway pressure (CPAP), as we wished to assess patients in whom the clinician selected a tidal volume, patients receiving chronic mechanical ventilation, and patients with missing or extreme values (> 99th percentile, to account for charting errors) of key data elements (ΔP, tidal volume, or C_RS_). Only the first episode of qualifying mechanical ventilation was analyzed for any given patient.

This study had two cohorts: patients without ARDS (primary cohort for analysis) and patients with ARDS (analyzed for known association of increased ∆P and tidal volume with mortality). We identified ARDS patients using the Berlin definition of ARDS [14] (Additional file 1: Table S1): (i) presence of an ARDS risk factor (trauma, pneumonia, sepsis, aspiration, shock, acute pancreatitis, or drug overdose) using claims data (Additional file 1: Table S2), (ii) ratio of partial pressure of arterial oxygen to fraction of inspired oxygen (P/F ratio) < 255 on at least 5 cm H_2_O PEEP (altitude corrected for Salt Lake City barometric pressure of 645 mmHg from a P/F ratio < 300) [2], (iii) pulmonary artery occlusion pressure < 18 mmHg if a right heart catheter was present, and (iv) chest radiograph (CXR) indicated bilateral infiltrates not due to effusion, atelectasis, or nodules [12, 15].

Considering the large number of patient records, we used natural language processing (NLP) of CXR reports to determine whether a patient met the fourth criterion of the Berlin definition of ARDS. We validated the NLP tool by testing it against a gold standard cohort of CXR reports read by radiologists on which the statistical NLP tool had not been trained, which included 1144 patients prospectively enrolled and confirmed as having ARDS for previous NHLBI studies at Intermountain Healthcare. The validated sensitivity, specificity, positive predictive value, and negative predictive value of the NLP tool were 0.81, 0.96, 0.86, and 0.94, respectively, with a corresponding F_1_ score of 0.83 and area under the receiver operating characteristic curve of 0.882.

### Exposure and outcome measures

We obtained data from Intermountain’s Electronic Data Warehouse, a carefully curated database integrating patient-level data from multiple clinical, billing, and administrative sources. Data on 30-day mortality (the primary outcome) were obtained using an existing linkage to Utah state death records. The primary exposure was the day 1 average ΔP of assessments performed every 2 h. The ventilator protocol instructs the respiratory therapist to measure of P_PL_ every 2 h using an end-inspiratory pause, often using a series of breaths averaged to get a value. We measured ΔP as P_PL_ − Set PEEP = ΔP, in cm H_2_O. The secondary exposures were day 1 average tidal volume and respiratory system compliance. Measured tidal volume was calculated using a time-weighted average of all measured tidal volumes obtained from the ventilator for day 1. Measurement of tidal volume was calculated from the ventilator as minute ventilation divided by respiratory rate. The Charlson Comorbidity Index was obtained from pre-admission billing diagnosis codes [16]. We used the lowest P/F ratio within 24 h after receipt of mechanical ventilation. APS score was calculated using data from the first 24 h before and after receipt of mechanical ventilation [17]. Ventilator-free days were calculated as days alive and free from ventilation out of 28 days.

### Statistical analysis

Our prespecified primary aim was to assess the association between ΔP and 30-day post-intubation mortality in non-ARDS patients. We performed logistic regression of ΔP vs. mortality while controlling for Acute Physiology Score (APS), Charlson Comorbidity Index (CCI), age, and P/F ratio. Our secondary aims included assessment of the association of set tidal volume and C_RS_ on mortality, ventilator-free days (out of 28 days), and ICU length of stay, separately, in non-ARDS patients. We also sought to confirm the expected associations of ΔP, set tidal volume, and C_RS_ on mortality, ventilator-free-days, and ICU length of stay in patients with ARDS. Primary and secondary analyses relied on complete-case analysis, so we conducted a sensitivity analysis and imputed missing values using multiple imputation with chained equations (MICE) [18]. Finally, we conducted an additional set of sensitivity analyses using generalized additive models (GAM) with tensor product smooths—a flexible extension of generalized linear models—to capture non-linear interactions, based on a recent simulation study of ARDS patients that suggested a non-linear relationship between tidal volume and mortality [19–21].

## Results

**Fig. 1:**
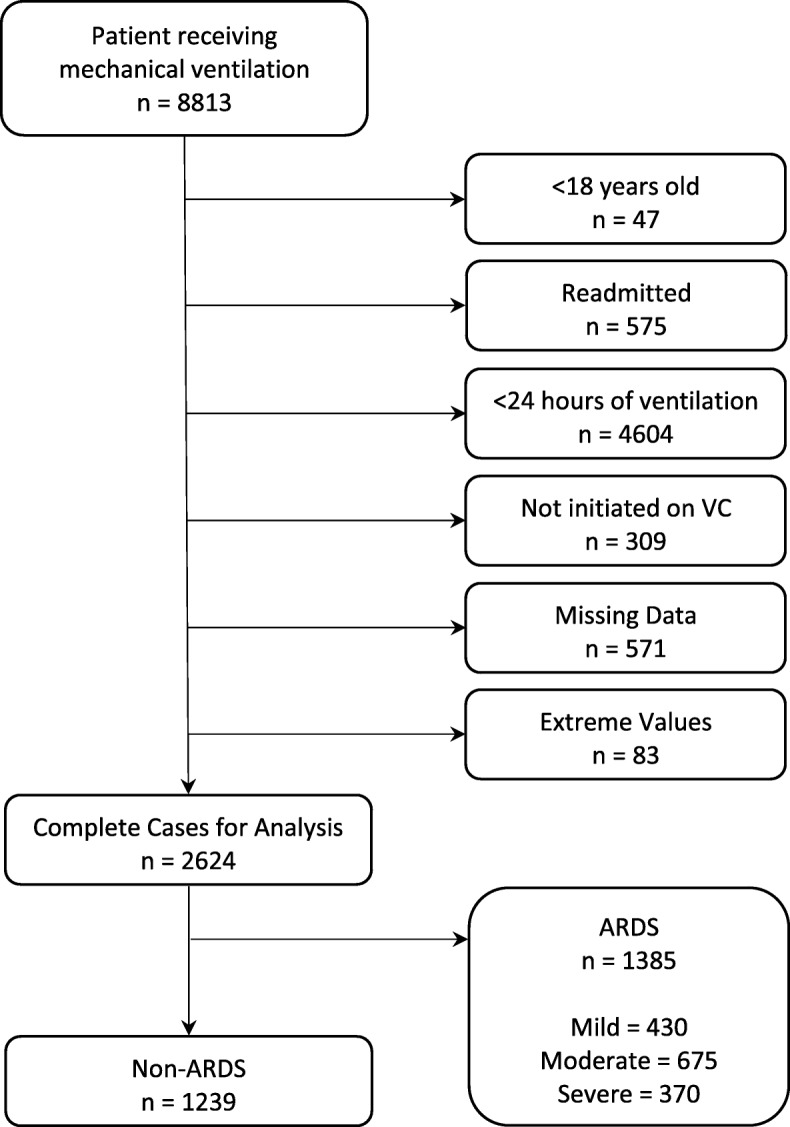
Patient inclusion diagram. VC: Volume-control ventilation. ARDS: acute respiratory distress syndrome. Extreme values are > 99th percentile of ΔP, tidal volume, or C_RS_, attributed to charting errors. Patients with missing values for ΔP, tidal volume, or C_RS_ were excluded from primary analysis, but used for sensitivity analyses after imputing missing values

From 2014 to 2015, we identified 8813 patients who started invasive mechanical ventilation at 17 ICUs from 12 Intermountain Healthcare hospitals. Volume-control ventilation was used in 91% of patients for initial ventilator settings. After exclusions, 2624 patients were eligible for enrollment, 53% with ARDS (*n* = 1385) and 47% without ARDS (*n* = 1239) (Fig. 1). Relevant patient characteristics are detailed in Table 1 and Additional file 1: Table S3.

**Table 1:** Demographic and clinical characteristics and clinical outcomes of patients with and without ARDS. Central tendencies are reported as median with interquartile ranges

	ARDS (*N* = 1385)	Non-ARDS (*N* = 1239)	*p* value
Baseline characteristics			
Age, years	61 (48–72)	60 (44–70)	0.007
Female, % (*n*)	42.1 (583)	42.0 (520)	0.98
Acute physiology score	25 (21–30)	23 (19–28)	< 0.001
Charlson Comorbidity Index	4 (2–6)	3 (1.5–6)	0.38
PaO_2_/FiO_2_ ratio, mm Hg	120 (83–170)	194 (121–284)	< 0.001
Mild hypoxemia (*n*)	340	703	
Moderate hypoxemia (*n*)	675	370	
Severe hypoxemia (*n*)	370	166	
PaO_2_, mm Hg	78 (66–95)	97 (76–136)	< 0.001
PaCO_2_, mm Hg	41 (35–48)	37 (32–43)	< 0.001
Ventilator parameters on day 1			
On PRVC, % (*n*)	74.0 (1025)	74.4 (922)	0.85
Set *V*_T_, normalized to PBW, mL/kg	6.3 (6.0–7.6)	6.6 (6.0–7.8)	0.04
For patients on PRVC, mL/kg	6.5 (6.0–7.8)	6.8 (6.1–7.9)	0.008
For patients on other VC, mL/kg	6.1 (6.0–6.8)	6.1 (6.0–6.9)	0.73
Measured *V*_T_, normalized to PBW, mL/kg	6.8 (6.2–7.8)	6.9 (6.3–7.8)	0.06
For patients on PRVC, mL/kg	6.9 (6.2–7.9)	6.9 (6.2–7.8)	0.03
For patients on other VC, mL/kg	6.8 (6.4–7.4)	6.7 (6.5–7.6)	0.32
Measured *V*_T_, mL	452 (386–516)	455 (392–520)	0.76
Respiratory rate, min^−1^	23 (19–28)	21 (18–25)	< 0.001
FiO_2_, %	45 (40–54)	42 (40–49)	< 0.001
PEEP, cm H_2_O	7.5 (5.0–9.9)	5.0 (5.0–8.0)	< 0.001
P_PL_, cm H_2_O	18.7 (16.0–21.9)	16.6 (14.0–19.6)	< 0.001
ΔP, cm H_2_O	10.8 (8.9–13.2)	10.0 (8.1–12.2)	< 0.001
C_RS_, mL/cm H_2_O	44.0 (34.1–55.4)	46.7 (37.8–59.2)	< 0.001
Clinical outcomes			
30-day mortality, % (*n*)	34.1 (472)	28.7 (356)	0.004
Ventilator-free days, out of 28 days	18 (0–24)	21 (0–25)	< 0.001
ICU length of stay, days	7.8 (4.0–13.5)	6.2 (3.3–12.1)	< 0.001

*C*_*RS*_ static respiratory compliance, *FiO*_*2*_ percent fraction of inhaled oxygen, *ΔP* driving pressure, *PaCO*_*2*_ pressure of dissolved arterial carbon dioxide, *PaO*_*2*_ pressure of dissolved arterial oxygen, *PEEP* positive end-expiratory pressure, *PRVC* pressure-regulated volume control ventilation mode, *PBW* predicted body weight, *P*_*PL*_ plateau pressure, *VC* volume control ventilation, *V*_*T*_ tidal volume

Patients with ARDS had higher mean acute physiology scores (25 vs 23, *p* value < 0.001) than non-ARDS patients but had similar comorbidities (CCI 4 vs. 3, *p* 0.38). Patients with ARDS had higher ΔP (11 vs 10 cm H_2_O, *p* < 0.001), higher PEEP (8 vs 5 cm H_2_O, *p* < 0.001) and lower set tidal volumes (6.3 vs 6.6 mL/kg PBW, *p* = 0.04). ARDS patients had fewer ventilator-free days (18 vs 21, *p* < 0.001), longer ICU length of stay (7.8 vs 6.2 days, *p* < 0.001), and had higher 30-day mortality (34.1% vs 28.7%, *p* = 0.004).

We observed that among ARDS patients, receipt of APC ventilation was associated with lower set tidal volumes (6.5 vs 6.8 mL/kg, *p* = 0.008) compared to tidal volumes of patients on other volume-controlled ventilatory modes. We also noted that delivered tidal volume was higher than set tidal volume (6.8 vs. 6.3 mL/kg, *p* < 0.001). This pattern was also observed in non-ARDS patients (6.9 vs 6.6 mL/kg, *p* = < 0.001). Patients receiving APC were more likely to have delivered tidal volume < 6.5 mL/kg (39.4% vs 28.1%, *p* < 0.001).

**Fig. 2:**
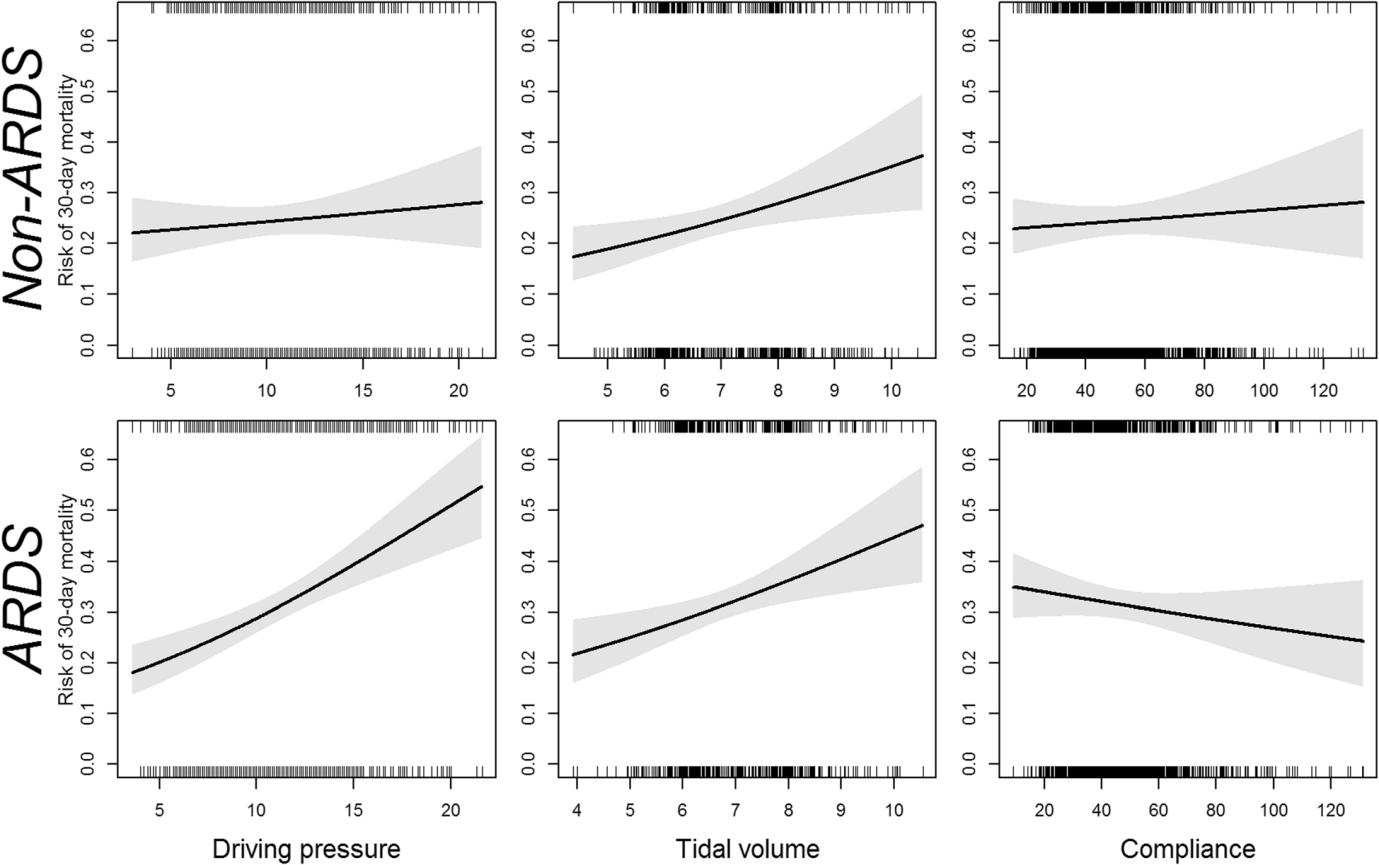
Estimated risk of 30-day mortality as a function of tidal volume, driving pressure, and respiratory system compliance among patients with and without ARDS, from generalized linear models. Hash marks indicated observed value for the exposures. Gray bands represent ± 2 standard errors

In our primary regression model of the association between set tidal volume and 30-day mortality among non-ARDS patients, tidal volume was associated with mortality (OR 1.22 per 1 mL/kg PBW increase in tidal volume, 95% CI 1.06 to 1.39, *p* = 0.010). By contrast, ΔP was not associated with mortality in non-ARDS patients. In ARDS patients, both ΔP and tidal volume were associated with mortality. We observed no adjusted association between C_RS_ and mortality in either cohort. Table 2 lists coefficient estimates, and Fig. 2 displays partial dependency plots. Results were unchanged after multiple imputation to allow inclusion of patients with missing data. As a post hoc analysis, we repeated the regression models, including covariates of spontaneous breathing (defined as a measured respiratory rate > set respiratory rate), we found that neither spontaneous breathing nor its interaction with driving pressure were significant in either patients with or without ARDS and that the inclusion of this interaction term in the models had no effect on the significance of other covariates.

**Table 2:** Adjusted association of tidal volume, driving pressure, and respiratory system compliance with 30-day mortality. Coefficient estimates from multivariable logistic regressions using generalized linear models (GLM). *p* values were adjusted for multiple hypothesis testing by limiting the false discovery rate (FDR) per the method of Benjamini and Hochberg [9]

Analysis	Group	Outcome	Exposure	OR (95% CI)	*p*	*p*_FDR_
Primary	Non-ARDS	30-day mortality	Driving pressure	1.02 (0.97 to 1.06)	0.463	
Secondary	Non-ARDS	30-day mortality	Tidal volume	1.22 (1.07 to 1.39)	0.003	0.01
Secondary	Non-ARDS	30-day mortality	Compliance	> 1 (0.99 to 1.01)	0.890	0.89
Confirmatory	ARDS	30-day mortality	Driving pressure	1.1 (1.06 to 1.14)	< 0.001	< 0.001
Confirmatory	ARDS	30-day mortality	Tidal volume	1.17 (1.04 to 1.31)	0.007	0.015
Confirmatory	ARDS	30-day mortality	Compliance	< 1 (0.99 to > 1)	0.314	0.472

Our generalized additive models demonstrated a nonlinear relationship but similar results between tidal volume and mortality in patients without ARDS, although the association persisted in patients with ARDS.

## Discussion

Our study is, to date, the largest evaluation of the association between ∆P and mortality in patients without ARDS. We did not observe a significant association between ∆P and mortality in non-ARDS patients, confirming findings from a previous single-center study [12]. This is in contrast to patients with ARDS where ΔP is significantly associated with mortality, both in our current study and other previous observational studies and a meta-analysis [10, 11, 22, 23].

The physiologic appeal of driving pressure is that it is essentially the tidal volume corrected for the C_RS_. Among patients with ARDS, who are much more susceptible to ventilator-induced lung injury, there may be some value in this construct. While older studies of ARDS demonstrated associations between C_RS_ and mortality [24], this association appears to be less prominent in the era of lung-protective ventilation [25]. This might explain why we found mortality in ARDS patients was associated with driving pressure and tidal volume to but not with compliance. For non-ARDS patients, while tidal volume is associated with mortality, we observed no relationship between C_RS_ and mortality, suggesting that the volutrauma depends on the relationship of tidal volume to total lung size but not to C_RS_ in non-ARDS patients. One possible explanation for this finding is that C_RS_ may not correlate with disease severity in non-ARDS patients, unlike ARDS patients, whose disease severity is inversely related to compliance. Obesity, ascites, thoracic, or abdominal surgery may affect respiratory compliance as well. There may also be some uncaptured confounding by indication among non-ARDS patients, as our generalized additive model for tidal volume in non-ARDS patients demonstrated a u-shaped curve with lower mortality at high tidal volumes. We observed comparable values of C_RS_ and ΔP between ARDS and non-ARDS patients, similar to prior studies [12, 22].

We did identify a significant association between lower tidal volume and improved survival in non-ARDS patients. This association, which supports the findings of other studies in non-ARDS patients [3, 6–8], is not consistent with a recent prospective randomized clinical trial [9]. Our non-linear model suggested a non-linear relationship between tidal volume and mortality in patients without ARDS. One possible explanation for this non-linear relationship might be confounding by indication, where healthier non-ARDS patients might be more likely to receive lower tidal volumes.

We are reassured that our data supports an association between tidal volume and mortality in ARDS patients and supports the recent meta-analysis confirming the association of ΔP and mortality in ARDS patients [11]. While the current state of evidence is not mature enough to recommend ΔP as a management strategy for ARDS [26], our data demonstrates no justification at this time to recommend it as a management strategy for non-ARDS patients.

We noted a large percentage of patients in our study cohort were managed with APC ventilation, which is a dual-controlled ventilation wherein the ventilator attempts to achieve tidal volume using a pressure-limited delivery format at the lowest possible airway pressure. While this mode of ventilation may decrease peak inspiratory pressures, no evidence exists that APC improves outcomes [27]. It is possible some of the differences we observed may be confounded by varying management strategies at different ICUs, some of which use APC more than others. The role of respiratory effort may affect the accuracy of ΔP in APC. In a passively ventilated patient, ΔP might be erroneously interpreted if the flow is not zeroed, while in a spontaneously breathing patient the ΔP might be affected by the changes in delivered volume associated with respiratory effort. Synchronization with the ventilator was not consistently recorded and therefore may affect the measurement of ΔP.

We also note a large percentage (48%) of patients were categorized as having ARDS. We suspect this bias is likely due to the enrichment of our study cohort by restricting to patients who have received mechanical ventilation to > 24 h. Patients with ARDS comprised only 14% of all patients with mechanical ventilation, but comprised a higher proportion of the study cohort due to the exclusion of patients who received mechanical ventilation < 24 h. One criticism of prior studies that have used radiographic reports to define ARDS is that they may have included patients who might have been less severe by including “ARDS” patients who were liberated from mechanical ventilation within 24 h [28]. While our NLP method was trained on radiology reports, we validated our tool against clinically confirmed cases of ARDS.

We further noted a low PiO_2_/FiO_2_ ratio among patients without ARDS. We speculate three reasons for the low ratio. First, the exclusion of people intubated for < 24 h enriches the population with patients who have significant gas exchange. Second, the increased application of high flow nasal cannula and non-invasive positive pressure ventilation likely prevent mechanical ventilation in many patients who would have a P/F ratio > 300. Last, the altitude of Salt Lake City will result in lower PiO_2_/FiO_2_ ratio (about 85% of what would be expected at sea level).

Our study has several limitations, including those typical of retrospective analysis. As clinicians were free to select ventilator settings, it is likely that there is some residual confounding not accounted for in our analyses. It is possible that the initial setting and mode may be more associated with outcome rather than ΔP. We analyzed day 1 ventilator settings, and it is possible that these settings may not be representative of the entire hospital course of ventilation. However, a recent study suggested that many initial ventilator settings were unlikely to be changed in the first few days [19]. Our ventilator protocol does not typically advise changes in ventilator mode aside from initiating weaning. The study patients were ventilated for over 24 h, which introduces bias compared to studying patients upon receipt of endotracheal intubation, including the increased proportion of ARDS and relatively high observed mortality in non-ARDS patients. The study ICUs are heterogenous with regard to patient population and with ventilator management preferences, allowing for the possibility of confounding. We did not capture whether patients were triggering the ventilator. In such patients, P_PL_ might be a poor surrogate of transpulmonary pressure. Similarly, we did not capture receipt of intravenous cistatricurium or prone positioning, interventions which might affect P_PL_ or ΔP. The cohort of non-ARDS patients comprises several different disease diagnoses, which may limit generalizability to other cohorts with different compositions of diagnoses. Our generalized additive model suggested the relationship between tidal volume and mortality is not linear in non-ARDS patients, which may decrease the validity of our primary analysis showing a linear association between tidal volume and mortality. Most study patients were managed with APC, meaning our findings may not be informative for other modes of ventilation.

## Conclusions

In a large retrospective analysis of critically ill non-ARDS patients receiving mechanical ventilation, we found tidal volume was associated with 30-day mortality, while ΔP was not. While this study supports the use of low tidal volume ventilation in all respiratory failure patients, there is insufficient evidence to justify managing critically ill non-ARDS patients by ΔP alone.

## Supplementary information

**Additional file 1.** Online Data supplement

## Data Availability

The datasets generated analyzed during the current study are available from the corresponding author on reasonable request.
