# Supplementary material for: Driving pressure is not associated with mortality in mechanically ventilated patients without ARDS
Source: Crit Care. 2019 Dec 27;23:424. doi: 10.1186/s13054-019-2698-9 (PMC6935179; doi:10.1186/s13054-019-2698-9)

Additional file

Sensitivity Analysis results

De-trended Q-Q plots featured residuals with rotated S-shapes (indicating that the tails of the fitted distributions were too heavy) and slightly negative slopes (indicating that the scales of the fitted distributions were slightly too high). Model calibration plots did not provide evidence of poor calibration, though the models did tend to slightly over-predict risk of mortality among those with the highest predicted risk. Receiver operating characteristic plots indicated moderate discriminatory ability (0.71 < AUC < 0.74), with sensitivity at optimal cutpoints ranging from 0.495 (model of ΔP and mortality in ARDS) to 0.730 (model of C_RS_ in non-ARDS), specificity ranging from 0.640 (model of C_RS_ in ARDS) to 0.831 (model of ΔP in ARDS), positive predictive value ranging from 0.457 to (model of C_RS_ in non-ARDS) to 0.605 (model of ΔP in ARDS), and negative predictive value ranging from 0.759 (model of ΔP in ARDS) to 0.858 (model of C_RS_ in non-ARDS).

Table S1. Berlin definition of acute respiratory distress syndrome (ARDS)^14^

| **Parameter** | **Criteria** | **Method for Determination** | |
| --- | --- | --- | --- |
| Timing | Within 1 week of a known clinical insult or new or worsening respiratory symptoms | | Patients intubated and mechanically ventilated within one week of hospital admission and not on chronic mechanical ventilation |
| Chest Imaging | Bilateral opacities—not fully explained by effusions, lobar/lung collapse, or nodules on a chest radiograph or computed tomography scan | | Natural language processing tool for review of chest radiograph reports to determine bilateral infiltrates |
| Origin of Edema | Respiratory failure not fully explained by cardiac failure or fluid overload  Need objective assessment (eg, echocardiography) to exclude hydrostatic  edema if no risk factor present | | Claims data (discharge ICD-9 and ICD-10 codes) determination of a risk factor for ARDS (trauma, sepsis, pneumonia, aspiration, shock, acute pancreatitis, or drug overdose) and exclusion of patients with a primary cardiac diagnosis, and electronic query of the EMR for placement of a pulmonary artery catheter excluding patients with a pulmonary artery occlusion pressure > 18 mm Hg if a right heart catheter was present |
| Oxygenation* | Mild: 170 mmHg < PF ≤ 255 mmHg  Moderate: 85 mmHg < PF ≤ 170 mmHg  Severe: PF ≤ 85 mmHg  (all with PEEP ≥5 cm H_2_O) | | Electronic data extraction from the Legacy and Cerner EMRs |

*All P/F ratios are adjusted for barometric pressure of Salt Lake City (645 mmHg)

CXR within 24 hours post-intubation, analyzed by NLP

ABG within 24 hours pos-intubation, lowest P/F ratio

PAOP within 24 hours of post-intubation

Table S2. Risk Factors for ARDS with a patient assigned to one risk factor if present by a prioritization scheme of trauma, pneumonia, sepsis, aspiration, shock, acute pancreatitis, or drug overdose.

| **Risk Factor for ARDS** | **Determination** |
| --- | --- |
| Trauma | Intermountain trauma registry |
| Pneumonia | ICD-9: 480-486, 487.0, 488.01, 488.11, 488.81  ICD-10: A48.1, J09.X1, J10.0, J11.0, J12-J18, J85.1 |
| Sepsis | Intermountain sepsis registry and  ICD-9: 008.8, 038.9, 041.12, 079.3, 079.89, 112.1, 530.4, 557.0, 590.80, 592.1, 593.9, 595.0, 599.0 682.9, 790.7, 785.52, 995.91, 995.92  ICD-10: A04.7, A41, J85.1, R65.2 |
| Aspiration | ICD-9: 507.0, 668.0  ICD-10: J69, O29.01, O74.0, O89.01 |
| Shock | ICD-9: 518.7, 669.1, 785.50, 785.59, 958.4, 995.0, 998.0  ICD-10: A48.3, O75.1, R57.1, R57.8, R57.9, T75.4, T78.0, T78.2, T79.4, T80.5, T81.1, T88.3 |
| Acute pancreatitis | ICD-9: 577.0  ICD-10: K85 |
| Drug overdose | ICD-9: 291.0, 291.81, 305.00, 960-979  ICD-10: F10.12, F10.22, F10.23, F11.12, F11.22, F13.12, F13.22, F14.12, F14.22, F15.12, F15.22, F16.12, F16.22, F18.12, F18.22, F19.12, F19.22, F19.23, T36-T50, Y90.8 |

Table S3. Primary reason for ARDS or reason for intubation. We categorized reason for ARDS or reason for intubation in Non-ARDS patients based on review of diagnostic claims data. For patients who might have met more than one criteria, we gave priority to some categories over others (listed in descending order).

|  | ARDS (n = 1385) | Non-ARDS (n = 1239) |
| --- | --- | --- |
| Trauma | 17% (237) | 16% (193) |
| Pneumonia | 38% (529) | 2% (27) |
| Sepsis | 32% (445) | 16% (197) |
| Aspiration | 7% (100) | 2% (19) |
| Shock | 3% (37) | 1% (15) |
| Pancreatitis | <1% (5) | <1% (2) |
| Overdose | 2% (32) | 7% (88) |
| Cardiothoracic Surgery |  | 15% (190) |
| Non-traumatic Brain Injury |  | 19% (232) |
| Cardiac Disorders |  | 10% (122) |
| Pulmonary Disorders |  | 3% (35) |
| Metabolic/Endocrine/  Renal Disorders |  | 6% (74) |
| Liver Failure |  | 2% (25) |
| Airway Protection/  Neuromuscular |  | 1% (10) |
| General Surgery |  | 1% (10) |

Table S4. Initial Mode of Mechanical Ventilation at Intermountain Hospitals in 2014 and 2015

| **Ventilation Mode** | **2014 Admits** | **2015 Admits** |
| --- | --- | --- |
| Volume Control Total | 3960 | 4070 |
| Pressure Regulated Volume Control | 2603 | 2724 |
| Other Volume Control | 1357 | 1346 |
| Pressure Control | 182 | 180 |
| APRV/Bi-level | 28 | 18 |
| Spontaneous CPAP and PS | 181 | 194 |
| **Total** | **4351** | **4462** |

CPAP = continuous positive airway pressure

PS = pressure support ventilation

APRV = airway pressure release ventilation

Figure S1, Partial dependence plots from multivariable logistic regression using generalized additive models (GAM). Solid black lines represent the estimated marginal effect of the exposure (ΔP, tidal volume, C_RS_) on mortality--that is, the estimated risk of mortality holding the covariates constant at their median value. Grey bands represent ±2 standard errors. Note the distinct non-linear marginal effect of tidal volume on mortality risk in patients without ARDS: an inverted U-shape.
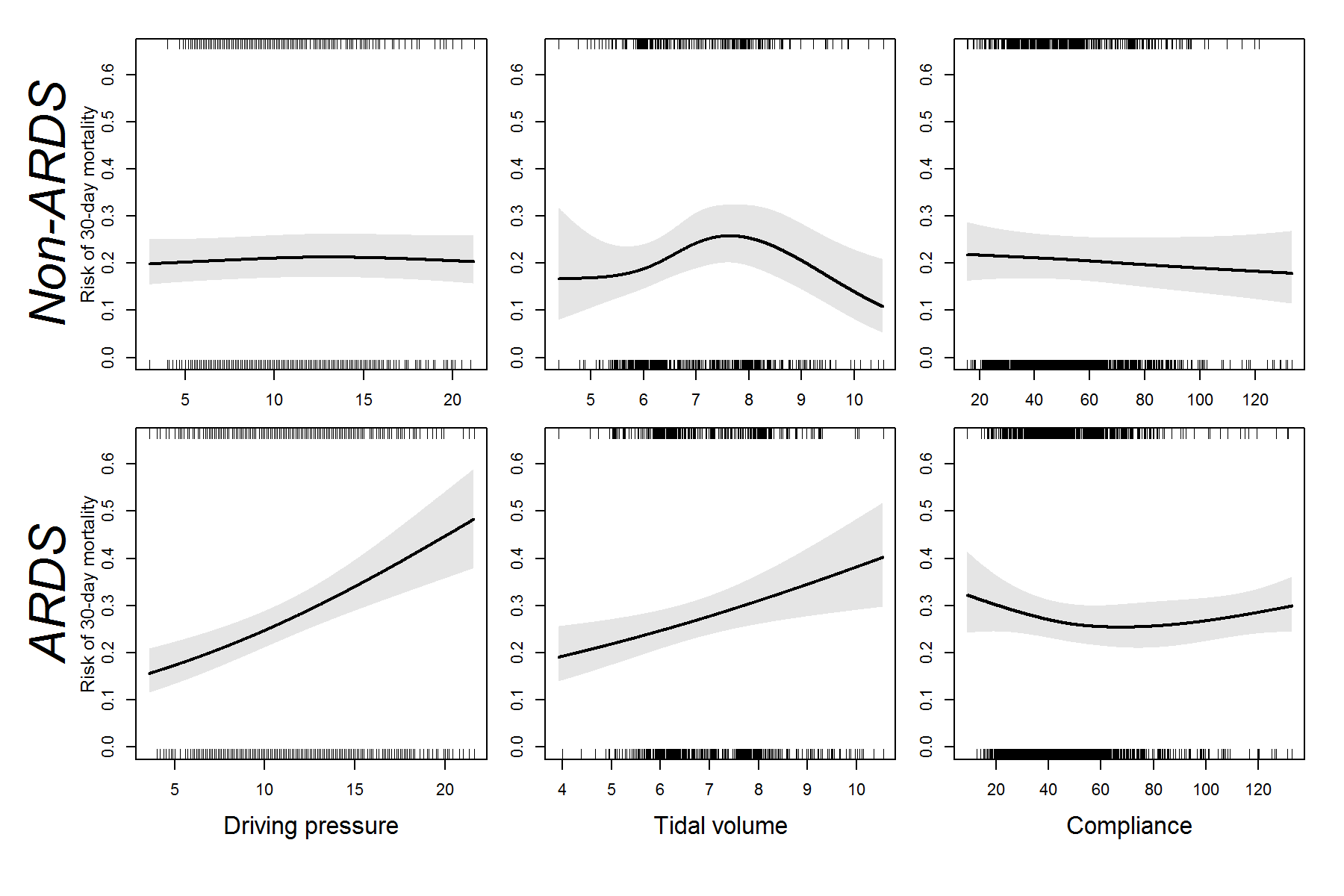

Supplement: Supplementary file 1 — Additional file 1. Online Data supplement [file 13054_2019_2698_MOESM1_ESM.docx]
